# Real-Time Monitoring of New Delhi Metallo-β-Lactamase Activity in Living Bacterial Cells by ^1^H NMR Spectroscopy**

**DOI:** 10.1002/anie.201308636

**Published:** 2014-01-23

**Authors:** Junhe Ma, Sarah McLeod, Kathleen MacCormack, Shubha Sriram, Ning Gao, Alexander L Breeze, Jun Hu

**Affiliations:** Discovery SciencesAstraZeneca Boston, Waltham, MA 02451 (USA); Infection Innovative MedicinesAstraZeneca Boston, Waltham, MA 02451 (USA); Discovery SciencesAstraZeneca Alderley Park, Macclesfield, Cheshire, SK10 4TG (UK)

**Keywords:** antibiotic resistance, drug discovery, meropenem, New Delhi metallo-β-lactamase, NMR spectroscopy

## Abstract

Disconnections between in vitro responses and those observed in whole cells confound many attempts to design drugs in areas of serious medical need. A method based on 1D ^1^H NMR spectroscopy is reported that affords the ability to monitor the hydrolytic decomposition of the carbapenem antibiotic meropenem inside *Escherichia coli* cells expressing New Delhi metallo-β-lactamase subclass 1 (NDM-1), an emerging antibiotic-resistance threat. Cell-based NMR studies demonstrated that two known NDM-1 inhibitors, L-captopril and ethylenediaminetetraacetic acid (EDTA), inhibit the hydrolysis of meropenem in vivo. NDM-1 activity in cells was also shown to be inhibited by spermine, a porin inhibitor, although in an in vitro assay, the influence of spermine on the activity of isolated NDM-1 protein is minimal. This new approach may have generic utility for monitoring reactions involving diffusible metabolites in other complex biological matrices and whole-cell settings, including mammalian cells.

Our knowledge of the structure and function of enzymes is obtained primarily through in vitro studies performed without the complete context of the physiological environment. As the scope of our understanding of individual components in cells becomes ever broader, we are motivated to explore protein structure and function in living cells in spite of the daunting challenges that arise from the complexity of the dynamic cellular environment and biological network.^1^ NMR spectroscopy is known as a noninvasive method for detecting substances. “In-cell” NMR technology, which aims to detect signals of overexpressed or introduced ^15^N-labeled proteins in living cells, has shown itself capable, in favorable circumstances, of providing valuable information about protein structure, folding, dynamics, and interaction with other cellular components in the native or seminative cellular environment.^2^

It has long been speculated that in-cell NMR could make useful contributions to the drug discovery process,^3^ particularly in the area of anti-infective research on serious bacterial pathogens. An example is provided by STINT-NMR, a technology based on the isotope labeling of only one partner in an intracellular protein complex by sequential expression.^4^ This technology has been applied to the screening of a small-molecule interactor library for compounds that can disrupt protein–protein interactions.^5^ However, the extent to which approaches based on the direct observation of the NMR resonances of proteins within the cellular milieu can be generally useful remains unclear. A number of factors conspire to render the observation of high-resolution spectra from intracellular globular proteins difficult: the elevated viscosity of the cytoplasm, macromolecular crowding effects that limit rotational and translational diffusion, and multiple transient protein–protein interactions driven by electrostatic interaction can all cause line broadening that can be so severe as to render all but the most mobile resonances of even relatively small proteins unobservable when these are overexpressed or introduced into the intracellular compartment.^6^, ^7^ Furthermore, the requirement to overexpress the protein(s) of interest substantially beyond their native expression levels can significantly perturb cellular function, thus calling into question the physiological relevance of the system under investigation. By contrast, monitoring small molecules such as drugs or metabolites after drug treatment in living cells should be relatively straightforward and feasible.^8^ In this case, the big hurdles for in-cell NMR, such as protein size limitation, isotope labeling, and overexpression, are avoided.

Our leading weapons against bacterial infections are β-lactams, which kill bacteria by inhibiting transpeptidases during bacterial cell-wall biosynthesis.^9^ One of the defense mechanisms that bacteria have evolved is to produce a variety of β-lactamases that disarm β-lactams by opening the cyclic amide ring.^9^, ^10^ Gram-negative bacterial pathogens such as *Escherichia coli* and *Klebsiella pneumoniae* that carry the New Delhi Metallo-β-lactamase subclass 1 (NDM-1) are called “superbugs” because they are resistant to most antibiotics and are challenging to treat.^11^ NDM-1 is a Class B β-lactamase.^9^, ^11^ Unlike Class A, C, and D β-lactamases, Class B β-lactamases possess a unique catalytic mechanism that utilizes Zn^2+^ ions in the ring opening of β-lactams.^12^

Carbapenems such as meropenem and imipenem, once trusted as a last resort to treat the most serious bacterial infections, can now be hydrolyzed by various β-lactamases, in particular, NDM-1 (Scheme 1).^13^ This reaction can be observed by using an in vitro NMR assay in which meropenem is treated with the purified NDM-1 enzyme (Figure 1 A). Meropenem is relatively stable for a long period of time in *E. coli* cells lacking carbapenemases (Figure 1 B). However, the drug is gradually degraded in the presence of *E. coli* cells carrying the NDM-1 enzyme (Figure 1 C). Despite the background signals from the cells and sample preparation (Figure S1 in the Supporting Information), the hydrolysis process can be clearly monitored by focusing on the ^1^H NMR signals from the methyl groups on meropenem. Under the experimental conditions employed (100 μM meropenem and a suspension of NDM-1 *E. coli* cells with an optical density at 600 nm (OD_600_) of 2.5 in sodium phosphate buffer), the intensity of the ^1^H signals from the methyl groups is about fivefold higher than from cells alone (Figure 1 B and C). In addition, the background ^1^H signals from the aromatic region are negligible (Figure S2), thus making this method generally applicable to common drugs and compounds in chemical libraries, of which approximately 80 % have aromatic groups.^14^ It is also a sensitive assay: even the terminal −N(CH_3_)_2_ protons (^1^H chemical shifts: δ=3.07 and 2.99 ppm, Figure 1) yield resolvable changes in chemical shift on ring opening. The viability of the NDM-1 *E. coli* cells was checked before and after the NMR experiments. The plating colony test shows that one hour of NMR measurements did not lead to any change in cell viability (Figure S3). To confirm that the enzymatic activity is from NDM-1 in the cells and to rule out the possibility that meropenem induces cell lysis and subsequent NDM-1 leakage into the medium, NDM-1 *E. coli* cells treated with meropenem were spun down and fresh meropenem was added to the supernatant to monitor the change in the meropenem ^1^H signals. Hydrolysis of meropenem was not observed (Figure S4), which demonstrates that the reaction occurs inside the cells. By contrast, when periplasmic proteins were released by treating NDM-1 *E. coli* cells with chloroform,^15^ hydrolysis of meropenem in the supernatant was observed by NMR spectroscopy (Figure S5). This result strongly supports the conclusion that the enzymatic reaction catalyzed by NDM-1 indeed occurs in the periplasmic space where most β-lactamases are known to reside.^12^

**Figure 1:**
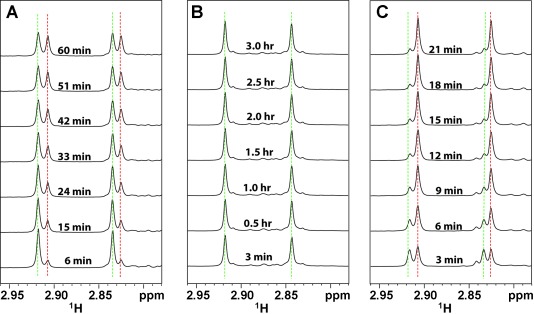
^1^H NMR spectra of meropenem hydrolysis in the presence of 5 nM purified NDM-1 enzyme (A), *E. coli* cells (OD_600_=10.0) without NDM-1 plasmid (B), and *E. coli* cells (OD_600_=2.5) expressing NDM-1 (C). All samples were prepared in 50 mM sodium phosphate at pH 7.0 with 10 % deuterated water. The hydrolysis of meropenem (100 μM) at different time points was monitored by focusing on the ^1^H NMR signals from the nitrogen-attached methyl groups (Scheme 1). The green and red dotted lines denote the signals of substrate and product, respectively.

**Scheme 1:**
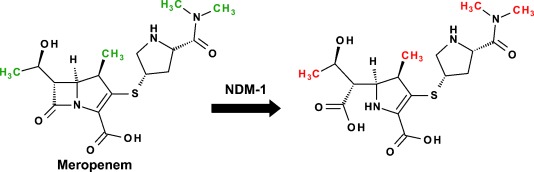
Hydrolysis of meropenem by the New Delhi Metallo-β-lactamase subclass 1 (NDM-1). The methyl groups of the substrate and product shown in green and red, respectively.

The stability of a few selected antibiotics (Figure S6) that display broad-spectrum antibacterial activities was compared in the presence of NDM-1 *E. coli* cells (Figure 2). It is known that NDM-1-positive strains are no longer susceptible to carbapenems such as meropenem and imipenem.^13^ These were readily hydrolyzed by the NDM-1 *E. coli* cells within an hour or so under our experimental conditions. Aztreonam, an exception among β-lactams, is not inactivated by metallo-β-lactamases.^16^, ^17^ Our results are consistent with this observation: no reduction in the NMR signals of aztreonam is seen in NDM-1 *E. coli* cells (Figure 2). It is reported that NDM-1-producing *Klebsiella pneumoniae* and *E. coli* are still susceptible to tigecycline.^16^, ^18^ Indeed, our data confirm that tigecycline is stable in the NDM-1 *E. coli* cells. Despite the fact that when using in vitro enzymatic assays, isolated NDM-1 enzyme shows moderate hydrolysis activity against cephalosporins such as cefotaxime and ceftazidime,^17^ no detectable change was observed within an hour when either drug was incubated with NDM-1 *E. coli* cells.

**Figure 2:**
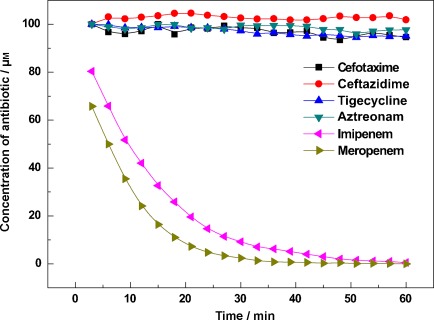
The stability of six antibiotics in the presence of the NDM-1 *E. coli* cells. The hydrolysis of six antibiotics was monitored through intensity changes in the methyl-group signals. The starting concentration of antibiotic was 100 μM and the cells were from the same batch with an OD_600_ of 2.5.

In order to protect β-lactam antibiotics from β-lactamase hydrolysis, a common approach is to combine them with β-lactamase inhibitors.^12^ For instance, ampicillin can be combined with sulbactam and amoxicillin with clavulanate. Unfortunately, there are as yet no useful metallo-β-lactamase (MBL) inhibitors available in clinical application.^12^, ^13^ Since the first case of NDM-1 was identified in 2008,^19^ NDM-1 has been rapidly disseminated across the continents and has been detected in a number of Gram-negative pathogens.^13^, ^18^ This poses a global epidemic threat that could result in a complete lack of available antibiotics for MBL-aggravated bacterial infections: “stormy waters ahead”, as Patel and Bonomo depicted in a recent review on carbapenemases.^13^

L-captopril and ethylenediaminetetraacetic acid (EDTA) are two known NDM-1 inhibitors.^20^, ^21^ In the crystallographic structure of NDM-1 in complex with L-captopril, the compound binds to the enzyme and its thiol group interacts with the two Zn^2+^ ions at the catalytic site.^22^ EDTA, on the other hand, may simply chelate out the Zn^2+^ ions that are critical for the activity of NDM-1. We examined meropenem hydrolysis by ^1^H NMR spectroscopy at different inhibitor concentrations. Figure 3 shows that the hydrolysis of meropenem can be inhibited by adding NDM-1 inhibitors to the cells. EDTA appears to have a much stronger inhibitory effect on meropenem hydrolysis than L-captopril. We determined the fifty percent inhibitory concentrations (IC_50_ values) of L-captopril and EDTA to be 175.0 and 1.6 μM, respectively. The IC_50_ values of L-captopril and EDTA against the NDM-1 enzyme are reported to be 72 and 0.4 μM, respectively.^20^, ^21^ We speculate that the bacterial cellular environment, which contains porins, efflux pumps, and the periplasmic space, may contribute to the lower inhibitory effect of these compounds under our whole-cell test conditions. However, the difference is not substantial.

**Figure 3:**
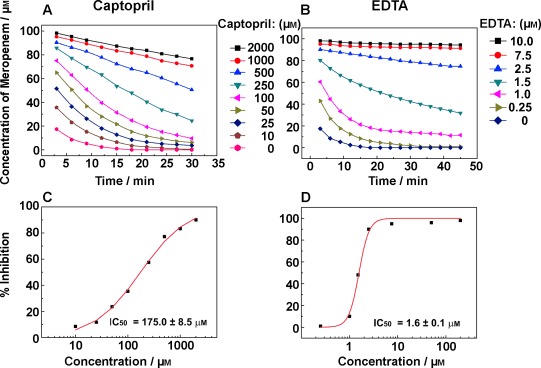
Inhibition of meropenem hydrolysis in the presence of NDM-1 *E. coli* cells by L-captopril (A) and EDTA (B) at various concentrations, and IC_50_ measurements for L-captopril (C) and EDTA (D). For each experiment, the NDM-1 *E. coli* cells (OD_600_=5.0) were first incubated with the inhibitor for 10 min and 100 μM meropenem was subsequently added.

A unique advantage of studying enzymatic reactions directly in cells is that other coupled biological components and functions can be explored as well. In this case, meropenem has to pass through the outer membrane barrier of *E. coli* cells through porins to be hydrolyzed by NDM-1 in the periplasm.^10^ Modulation of the open/closed state of porins should therefore influence the enzymatic reaction in the cell. Indeed, Figure 4 A shows that the rate of meropenem hydrolysis is dependent upon the concentration of a porin inhibitor, spermine.^23^ By contrast, spermine does not inhibit the hydrolysis of meropenem by the NDM-1 enzyme in vitro (Figure 4 B). It is likely that spermine reduces the permeation of meropenem into the periplasm, which limits its access to NDM-1 and consequently results in a reduction of the rate of meropenem hydrolysis.

**Figure 4:**
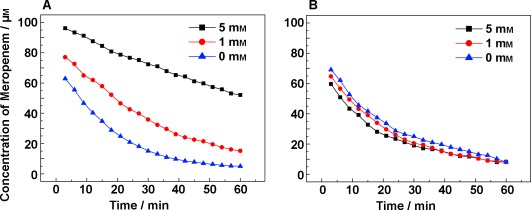
The influence of spermine (a porin inhibitor) on meropenem hydrolysis by NDM-1 *E. coli* cells (A) and isolated NDM-1 enzyme (B). The NDM-1 *E. coli* cells (OD_600_=1.0) and 25 nM purified NDM-1 enzyme were first incubated with 0, 1, 5 mM spermine for 10 min. ^1^H NMR spectra were recorded at 25 °C after the addition of 100 μM meropenem.

In conclusion, we have shown that ^1^H NMR spectroscopy is a simple and powerful method to study bacterial enzyme functions in their native cellular environment. Although we have chosen to exemplify the technique with bacterial cells, we believe the approach has the potential for more general application to many types of complex biological matrices, including eukaryotic cell systems. The methodology could also be applied to challenging proteins, for instance, integral membrane proteins. The procedures required for the preparation of membrane proteins for in vitro studies (e.g. detergent solubilization, purification, refolding and reconstitution in membrane-mimicking lipid bilayers) very often result in significant loss of activity. By contrast, in our proposed method, membrane protein function may be assayed directly in the native membrane milieu and complex sample preparation is not required. The functional data obtained from NMR measurement is closely related to biological function in the physiological environment. Our NDM-1 study can be applied as a target-based whole-cell screen to search for potent NDM-1 inhibitors. Unlike common phenotypic whole-cell screening, the NMR approach does not require post-screen target deconvolution, because the activity of the target enzyme is monitored through direct spectroscopic observation of substrate/product turnover. Moreover, compound access to the target within the cell is implicitly accounted for in the screening. This novel screening approach is currently under development for other applications.
