# Supplementary material for: Real-Time Monitoring of New Delhi Metallo-β-Lactamase Activity in Living Bacterial Cells by 1H NMR Spectroscopy
Source: Angew Chem Int Ed Engl. 2014 Jan 23;53(8):2130–3. doi: 10.1002/anie.201308636 (PMC4232273; doi:10.1002/anie.201308636)
Supplement: Supplementary file 1 [file anie0053-2130-sd1.pdf]

Angewandte Chemie
Eine Zeitschrift der Gesellschaft Deutscher Chemiker

Supporting Information

© Wiley-VCH 2014

69451 Weinheim, Germany

Real-Time Monitoring of New Delhi Metallo-β-Lactamase Activity in Living Bacterial Cells by ^{1}H NMR Spectroscopy**

Junhe Ma, Sarah McLeod, Kathleen MacCormack, Shubha Sriram, Ning Gao, Alexander L. Breeze, and Jun Hu**

anie_201308636_sm_miscellaneous_information.pdf

CONTENTS:

1. Materials and methods

1-1. Reagents
1-2. NDM-1 enzyme expression and purification
1-3. Construction of ARC4670 and ARC4671 (with and without the NDM-1 plasmid) *E. coli* cells
1-4. *E. coli* cells sample preparation
1-5. Plating colony tests for NDM-1 *E. coli* cells
1-6. Releasement of periplasmic proteins
1-7. ^{1}H NMR measurements and analyses
1-8. IC_{50} measurement

2. Supporting figures

3. References

1. Materials and methods

1-1. Reagents

All antibiotics and inhibitors used in this study were purchased from Sigma-Aldrich Corporation.

1-2. NDM-1 enzyme expression and purification

For protein overproduction, the plasmid was transformed into BL21(DE3) (EMD Chemicals, Gibbstown, NJ) and plated on Luria-Bertani (LB) medium containing 50 µg/ml kanamycin at 37 °C overnight. A single colony of BL21(DE3)/pJT1080 was inoculated into a 100-ml culture of LB containing 50 µg/ml kanamycin and grown overnight at room temperature. The overnight culture was diluted to OD_{600} = 0.1 in 4 x 1L of LB containing 50 µg/ml kanamycin and grown at room temperature, with aeration, to mid-logarithmic phase (OD_{600} = 0.6). IPTG was then added to a final concentration in each culture of 0.5 mM. After overnight induction at room temperature, the cells were harvested by centrifugation at 5,000 x g for 15 min at 25 °C. Cell paste was stored at –20 °C.

The frozen cell paste was suspended in 50 ml of Lysis Buffer [50 mM HEPES, pH 7.5, 50 µM $ZnSO_4$, 5% glycerol, 1 Protease inhibitor cocktail tablet (Roche Diagnostics, Indianapolis, IN)]. Cells were disrupted by passing them twice through a French press operated at 18,000 psi, and the crude extract was centrifuged at 30,000 rpm (45Ti rotor, Beckman-Coulter, Brea, CA) for 30 min at 4ºC. The supernatant was loaded at a flow rate of 2.0 ml/min onto a 20 ml Q-Sepharose HP (HR16/10) column (GE Healthcare Life Sciences, Piscataway, NJ) pre-equilibrated with Buffer A (50 mM HEPES, pH 7.5, 50 µM $ZnSO_4$, 5% glycerol). The column was then washed with Buffer A, and the protein was eluted by a linear gradient from 0 to 1 M NaCl in Buffer A. Fractions containing NDM-1 were pooled, and solid $(NH_4)_2SO_4$ was added to a final concentration of 1 M. The sample was applied at a flow rate of 2.0 ml/min to a 20 ml Phenyl Sepharose HP (HR16/10) column (GE Healthcare Life Sciences) pre-equilibrated with Buffer B [50 mM HEPES, pH 7.5, 50 µM $ZnSO_4$, 5% glycerol, 1M $(NH_4)_2SO_4$]. The column was washed with Buffer B, and the protein was eluted by a linear gradient from 1 to 0 M $(NH_4)_2SO_4$ in Buffer A. Fractions containing NDM-1 were pooled and concentrated to 5 ml by Amicon® Ultracel-10K (Millipore, Billerica, MA). The 5 ml sample was applied at a flow rate of 1.5 ml/min to a 120 ml Sephacryl S-100 (HR 16/60) (GE Healthcare Life Sciences) column pre-equilibrated with Buffer C (50 mM HEPES, pH 7.5, 50 µM $ZnSO_4$, 10% glycerol, 150 mM NaCl). The fractions containing NDM-1 were pooled and concentrated by Amicon® Ultracel-10K (Millipore). The protein concentration was determined by the method of Bradford and characterized by SDS-PAGE analysis and analytical LC-MS. The protein was stored at –80 ºC.

1-3. Construction of ARC4670 and ARC4671 (with and without the NDM-1 plasmid) *Escherichia coli* cells

ARC4670 is a derivative of *Escherichia coli* strain that has an in-frame deletion on the chromosome of *waaP*.[1] The *waaP* gene deletion was first constructed in the *E. coli* strain BW25113 containing the lambda Red recombination system on plasmid pKD46 as previously described.[2] The deletion of *waaP*, which was replaced by a kanamycin resistance gene flanked with FRT sites, was then moved by P1 phage transduction to W3110.[3] The kanamycin resistance gene was then excised from the chromosome using

the FLP recombinase expressed from plasmid pCP20.[2] The plasmid pBBR1MCS-NDM-1, which contains NDM-1 expressed from plasmid PBBR1MCS-2, was then transformed into this resulting strain by electroporation, selecting on LB Kanamycin 25 µg/ml.[4] To construct plasmid pBBR1MCS-NDM-1, NDM-1 was amplified by PCR from the *E. coli* clinical isolate ARC3600 using primers (5'-ACCCATATGGAATTGCCCAATATTATG-3') and (5'-GGTGGATCCTCAGCGCAGCTTGTCGGC-CATG- 3'). The PCR product was then cloned into the *NdeI* and *BamHI* restriction sites of pBBR1MCS-2. ARC4671 is the same strain background as ARC4670, except it carries the empty vector pBBR1MCS-2 instead of pBBR1MCS-NDM-1.

1-4. *E. coli* cells sample preparation

5 µl of NDM-1 cell seeds were inoculated into 5 mL LB media in the presence of 25 µg/mL kanamycin and grown with shaking at 37 °C for overnight. Cell cultures were centrifuged at 2500 RPM for 20 min at 4 °C. The cell pellets were resuspended in 1 mL buffer (50 mM sodium phosphate in 90%H_2O/10%D_2O, pH 7.0). The cell suspension was centrifuged again and the supernatant was discarded. This process was repeated at least three times. The final OD_{600} of the cell suspension was calculated by multiplying the measured OD_{600} with the fold of dilution (the OD_{600} readings were adjusted in the range of 0.1 ~ 0.6 for accuracy). 500 µL of final cell suspensions in phosphate buffer were placed in 5 mm NMR tubes for data acquisition. Supernatants from the cell suspension were collected by centrifugation (Eppendorf, model 5418, 13,500 RPM for 15 min), then filtered by 0.22 µM filter.

1-5. Plating colony tests for NDM-1 *E. coli* cells

Plating colony tests were conducted as previously described.[5] First, at the bottom of an LB agar selective plate containing 25 µg/ml kanamycin, six sections were drawn using a marker. For the stock suspensions of NDM-1 *E. coli* cells, serial 10^2, 10^4, 10^5, 10^6, 10^7 and 10^8 –fold dilutions were performed and 10 µl drops of each dilution plated on the marked section of the LB plate. The drops were allowed to dry on the plate and the plates incubated at 37 °C overnight. The section containing 10 or so colonies was then identified, the number of colonies counted, and the numbers in equivalent sectors for samples under different conditions compared.

1-6. Release of periplasmic proteins

The release of periplasmic proteins in NDM-1 *E. coli* cells followed the previously published protocol using chloroform (see reference 15 in the text). The NDM-1 *E. coli* cell pellet was suspended in 20 µl of 50 mM Tris buffer (pH 8.0), followed by the addition of 20 µl deuterated chloroform. The sample was vortexed briefly. Then, 180 µl of Tris buffer (pH 8.0) was added and the cells were incubated on ice. After 30 minutes, the sample was centrifuged and the supernatant was passed through a 0.22 µM filter. Finally, 300 µl of 50 mM sodium phosphate buffer (pH 7.0) and meropenem (final concentration: 100 µM) were added. The hydrolysis of meropenem was monitored by ^{1}H NMR.

1-7. ^{1}H NMR measurements and analyses

All spectra were acquired on Bruker Avance 600 MHz or500 MHz spectrometers equipped with TCI or QNP pulsed-field z-gradient Cryoprobes, respectively. For 1D ^{1}H NMR experiments, a sweep width of 9615.385 Hz (600 MHz) or 7002.801 (500 MHz) was used. All spectra were acquired with 64 scans. The temperature was kept constant at 25 °C if not noted otherwise. Water suppression was achieved by means of the excitation sculpting scheme; the water-selective 180° sinc-shaped pulse was 3 ms long.[6] The free induction decay was collected in 32K data points. The residual water resonance was suppressed by application of a time-domain deconvolution digital filter. A 1 Hz exponential line broadening function

was applied prior to Fourier transformation. NMR spectra were processed and analyzed with Topspin 3.2. All spectra in a given series are plotted at the same scaling ratio.

1-8. IC_{50} measurements and analyses

The IC_{50} measurements by NMR spectroscopy follow the method introduced by Dalvit et al.[7] After 21 minutes of the NMR experiment, the percentage of inhibition is calculated as shown below (Equation 1):

$$\% \text{ Inhibition} = 100\times[1 - ([S_{TOT}] - [S_w])/ ([S_{TOT}] - [S_{w/o}])] \qquad \text{[Equation 1]}$$

where $[S_w]$ and $[S_{w/o}]$ are given by the intensities of the substrate signal (3.07 ppm of meropenem in this case) in the presence and absence of the inhibitor, respectively. $[S_{TOT}]$ is the sum of $[S_{w/o}]$ and $[P_{w/o}]$ or $[S_w]$ and $[P_w]$, where $[P_w]$ and $[P_{w/o}]$ are the intensities of the product signal (3.06 ppm of hydrolyzed meropenem in this case) in the presence and absence of the inhibitor, respectively. IC_{50} can be obtained by fitting Equation 2:

$$\% \text{ Inhibition} = 100 \times [1 - 1/(1 + ([I]/IC_{50})^n)] \qquad \text{[Equation 2]}$$

where [I] is the concentration of the inhibitor and n is the cooperativity factor.

2. Supporting figures

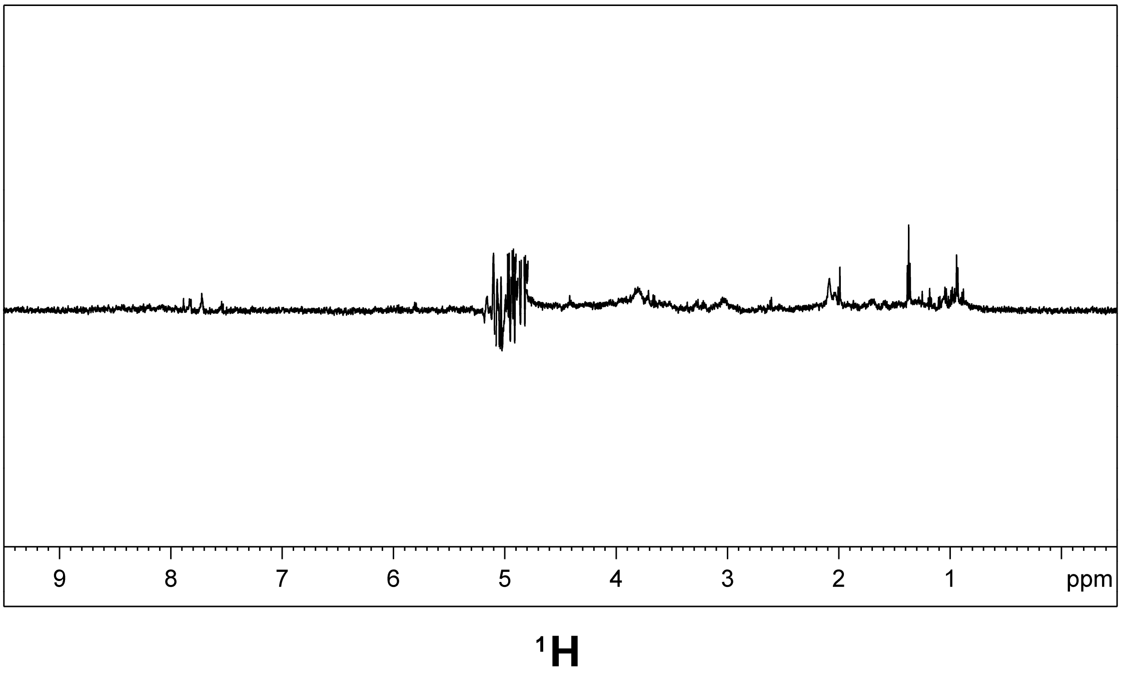

Figure S1. ^{1}H NMR spectrum of a suspension of NDM-1 *E. coli* cells (OD_{600} = 2.5) in 50 mM sodium phosphate buffer.

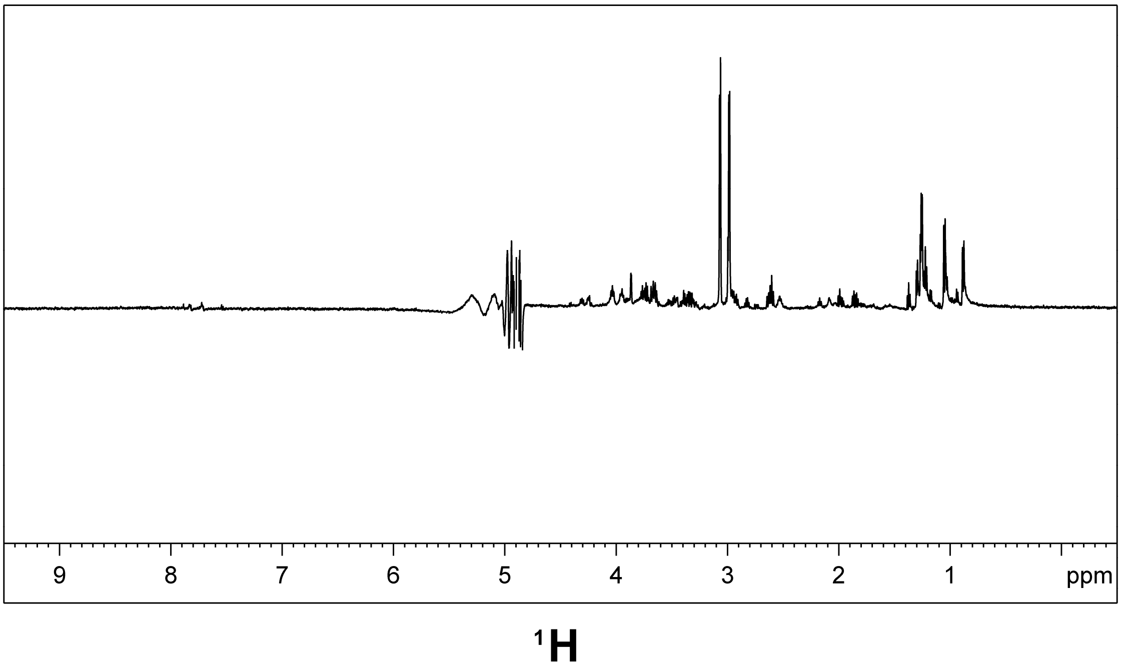

Figure S2. ^{1}H NMR spectrum of 100 μM meropenem in the presence of NDM-1 *E. coli* cell suspension (OD_{600} = 2.5) after three minutes of the reaction.

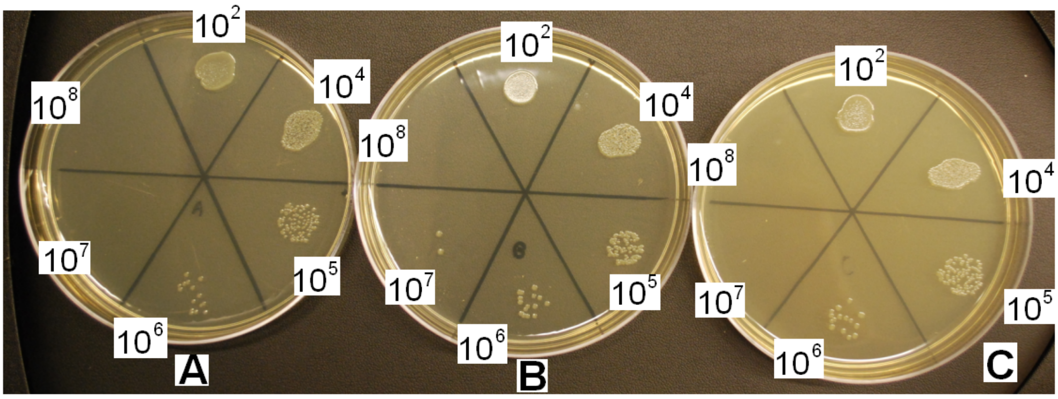

Figure S3. Plating colony tests to examine NDM-1 *E. coli* cell viability before and after NMR experiements. All NDM-1 *E. coli* cells in these tests were from the same batch. The fold of dilution is labeled on each section of the LB/kanamycin plates. NDM-1 *E. coli* cells without the treatment of meropenem were plated on plate A. Cells with the addition of_100 µM meropenem before and after one-hour NMR experiments were plated on plate B and C, respectively. The section labeled with 10^6 fold dilution is used for the study of cell viability. There are 16, 15 and 15 single colonies on plate A, B and C, respectively. This data demonstrate that cells were alive during the NMR experiments and there is little differences of cell viability before and after the experiments.

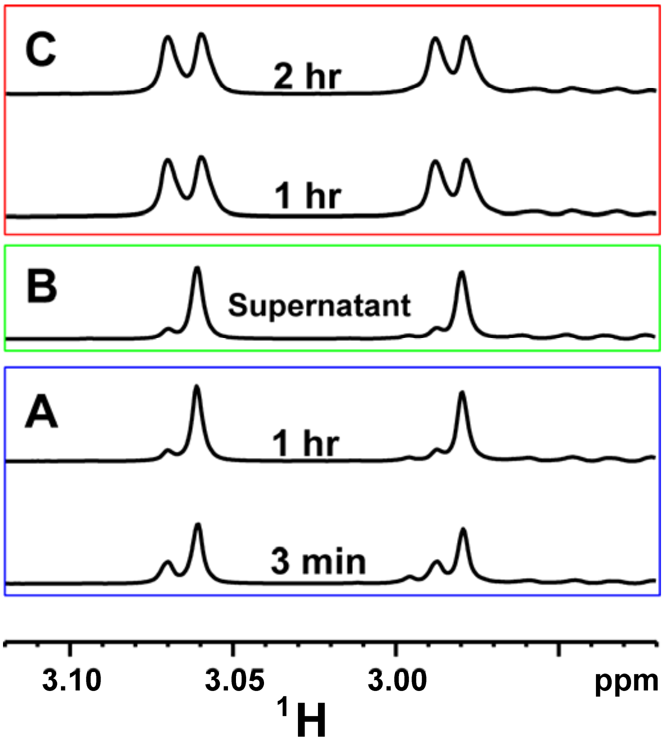

Figure S4. NDM-1 catalyzed hydrolysis occurs inside *E. coli* cells. A) NMR spectra of NDM-1 *E. coli* cells (OD_{600} = 2.5) with the addition of 400 µM meropenem; B) NMR spectra of the supernatant of sample A after 1 hour incubation in the magnet. C) NMR spectra of sample B with addition of another 400 µM fresh meropenem. All samples were prepared in 50 mM sodium phosphate at pH 7.0 containing 10% deuterated water.

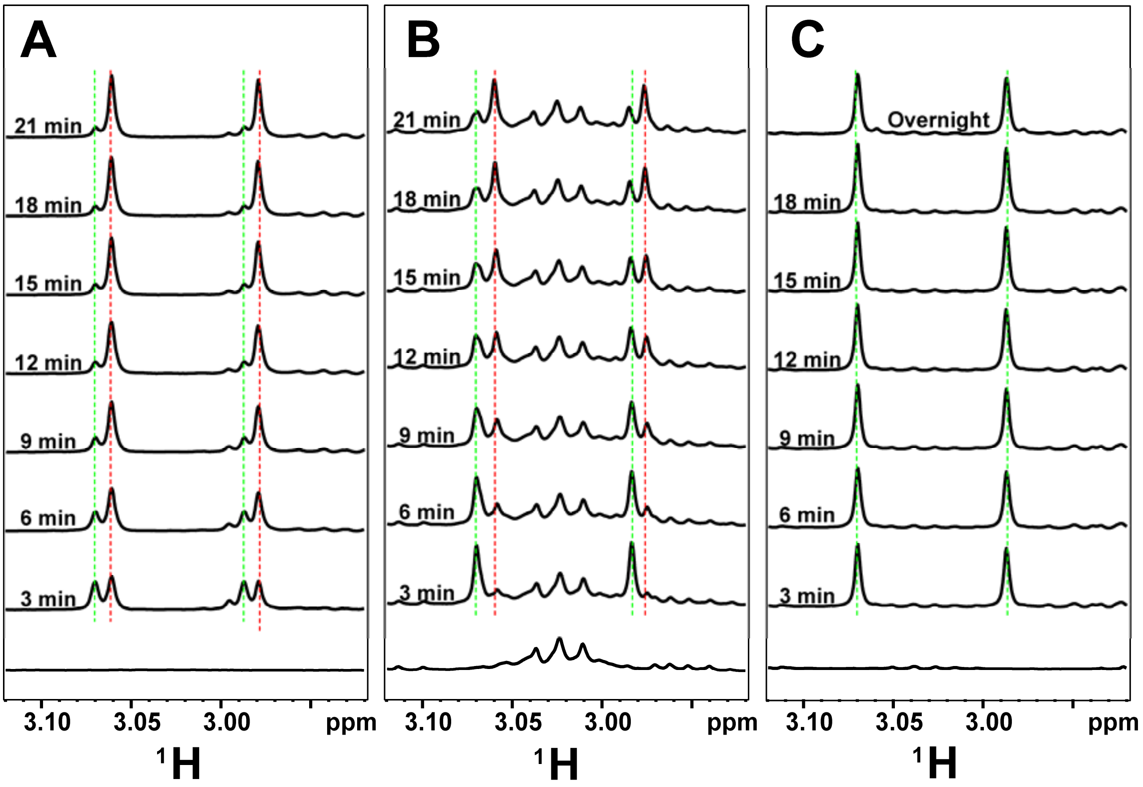

Figure S5. Hydrolysis of meropenem by NDM-1 released from the periplasm of the NDM-1 *E. coli* cells. A) NMR spectra of NDM-1 *E. coli* cells with 100 μM meropenem; B) NMR spectra of 100 μM meropenem added to the supernatant of NDM-1 *E. coli* cells treated with deuterated chloroform; C) NMR spectra of the supernatant of NDM-1 *E. coli* cells with 100 μM meropenem. The NMR spectra of samples before the addition of meropenem are shown at the bottom as references. The green and red dotted lines denote the signals of substrate and product, respectively.

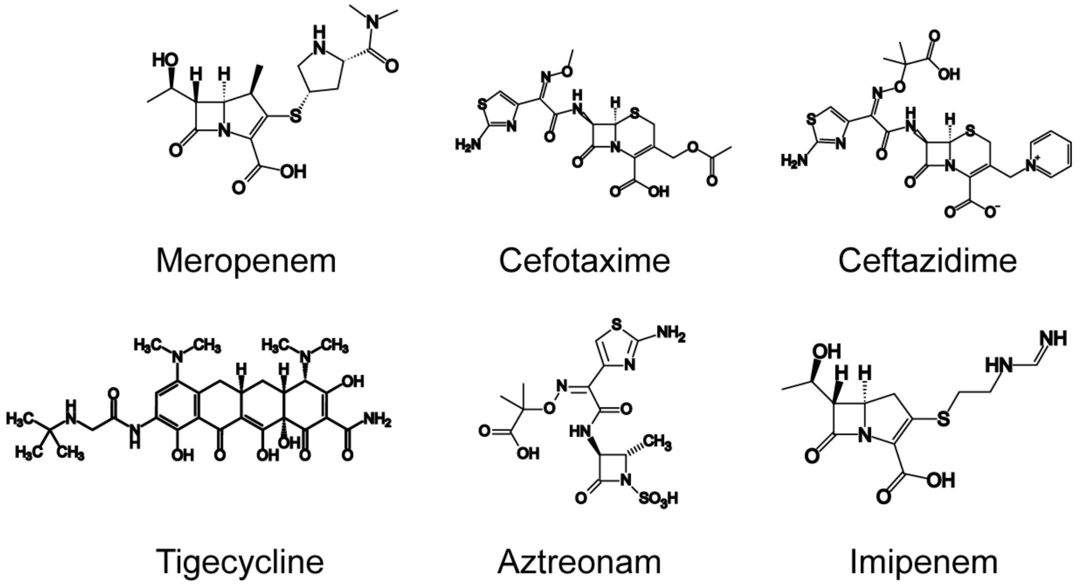

Figure S6. Chemical Structures of antibiotics selected for stability test.

3. References:

[1] K. Hayashi, N. Morooka, Y. Yamamoto, K. Fujita, K. Isono, S. Choi, E. Ohtsubo, T. Baba, B. L. Wanner, H. Mori, T. Horiuchi, *Mol. Syst. Biol.* **2006**, *2*, 2006-0007.

[2] K. A. Datsenko, B. L. Wanner, *Proc. Natl. Acad. Sci. U S A.* **2000**, *97*, 6640-6645.

[3] L. C. Thomason, N. Costantino, D. L. Court, *Curr. Protoc. Mol. Biol.* **2007,** Chapter 1, Unit 1.17.

[4] M. E. Kovach, P. H. Elzer, D. S. Hill, G. T. Robertson, M A. Farris, R. M. 2nd Roop. K. M. Peterson, *Gene* **1995**, *166*, 175-176.

[5] K. E. Robinson, P. N. Reardon, L. D. Spicer, *Protein NMR Techniques, Methods in Molecular Biology* Springer Science+Business Media **2012**; pp261-277.

[6] K. S. Scott, J. Keeler, T. L. Hwang, A. J. Shaka, *J. Am. Chem. Soc.* **1995**, *117*, 4199-4200.

[7] C. Dalvit, E. Ardini, G. P. Fogliatto, N. Mongelli, M. Veronesi, *Drug Discov. Today* **2004**, *9*, 595-602.
